# Evaluating trade-offs between COVID-19 prevention and learning loss: an agent-based simulation analysis

**DOI:** 10.1098/rsos.231842

**Published:** 2025-04-23

**Authors:** Kenneth Chen, Eva A. Enns

**Affiliations:** ^1^ Harvard University, Cambridge, MA, USA; ^2^ Division of Health Policy and Management, University of Minnesota School of Public Health, Minneapolis, MN, USA

**Keywords:** COVID-19, mathematical modelling, learning loss

## Abstract

The COVID-19 pandemic presented significant challenges in educational settings. Schools implemented a variety of COVID-19 mitigation strategies, some of which were controversial due to potential disruptions to in-person learning. We developed an agent-based model of COVID-19 in a US high school setting to evaluate potential trade-offs between preventing COVID-19 infections versus avoiding in-person learning loss under different mitigation policies in a post-Omicron context. Mitigation policies included isolation alone and in combination with quarantine of exposed students, weekly testing of all students or testing of exposed students (‘test-to-stay’) under different scenarios of mask use and booster dose uptake. Outcomes were simulated over an 11 week trimester. We found that requiring a full 5 or 10 day quarantine of exposed students reduced COVID-19 infections by five to sevenfold relative to isolation alone, but at a cost of nearly 40% learning days lost. Test-to-stay achieved nearly the same level of infection reduction with lower levels of learning loss. Weekly testing also reduced COVID-19 infections but was less effective and incurred higher learning loss than test-to-stay. Universal masking and increased vaccination not only reduced infections at no cost to learning but also synergized with other strategies to reduce trade-offs.

## 1. Introduction

The COVID-19 pandemic, driven by transmission of the SARS-CoV-2 virus, presented significant challenges and disruptions to US educational settings. The early phase of the pandemic saw widespread K-12 school closures in the spring of 2020 affecting up to 55 million students in 124 000 schools [1]; the school years that followed consisted of a mix of in-person and virtual instruction with varying layers of mitigation in place in response to ever-changing guidance and epidemiological conditions. Throughout the pandemic, school policy decisions have been the subject of debate, with COVID-19 precautions framed as being at odds with consistent in-person learning [2,3]. In August 2022, the United States Centers for Disease Control and Prevention (CDC) ended many of their recommendations for COVID-19 mitigation in schools, including quarantine after exposure, routine asymptomatic testing and cohorting [4]. Some praised these policy changes for keeping students in the classroom and avoiding lost in-person instruction [5]; however, others warned that removing mitigations would risk larger outbreaks that could threaten the health of students and their families and cause even further disruption to students’ learning [6].

Mathematical modelling is a useful tool for comparing outcomes under different policy scenarios and identifying trade-offs in potentially competing objectives, such as reducing SARS-CoV-2 infections in schools while also minimizing learning loss. Agent-based simulation is a modelling technique that simulates the interactions of individual, autonomous agents. Since being first introduced in 1971 to model residential self-segregation [7,8], it has been applied to a range of biological, economic, social and physical domains [9,10], including epidemiology [11]. Before and during the 2009 H1N1 pandemic, agent-based models were used to inform pandemic preparedness and response strategies, where their granularity could provide advantages over traditional differential equation models of infectious disease spread [12–14]. Traditional models, which typically assume homogeneous and perfect mixing, are still widely used to model infectious diseases including COVID-19 [15,16] and remain crucial tools for public health officials. However, agent-based models can more realistically represent environments where heterogeneity and network structure are central to disease transmission and interventions, such as a school [17].

Several modelling studies have evaluated COVID-19 mitigations in schools [18–23], but only a small number have considered learning loss. These modelling studies found that quarantining entire classrooms leads to a high number of in-person learning days lost, but that regular testing could achieve similar reductions in infection risk with much less learning loss, particularly when community transmission levels are low [20–23]. These findings were consistent across educational contexts (primary versus high school) and multiple countries. However, these studies were done in the context of the original SARS-CoV-2 strain. The emergence of the highly infectious Omicron variants has significantly changed SARS-CoV-2 infection dynamics and, in turn, the impact of mitigation policies. In particular, the shorter Omicron incubation period makes it more challenging to identify infectious individuals early with typical routine testing frequencies (e.g. weekly), but also potentially allows for shorter quarantine period (e.g. 5 days), which would reduce the learning loss burden of some mitigation strategies.

To expand our understanding of COVID-19 mitigation and learning loss trade-offs, specifically in the context of the more infectious Omicron variants, we developed an agent-based model to simulate the spread of COVID-19 in a high school setting. We used the model to evaluate the impact of different combinations of COVID-19 mitigation policies on both SARS-CoV-2 infections and in-person learning days lost. Our study further contributes to new understanding of COVID-19 mitigations and learning loss by evaluating how school community behaviours, such as mask use and vaccine uptake, impact health and educational outcomes and the potential trade-offs between them. Because we evaluate policies that apply to students in a heterogeneous manner and are affected by the unique contact structure of a school, we used a stochastic agent-based simulation model for this analysis.

## 2. Methods

We developed an agent-based model to simulate the transmission of the SARS-CoV-2 virus between students in a high school setting in South Washington County, a suburban school district in Minnesota. The model was calibrated to reproduce observed weekly case counts during the delta variant wave from September to November 2021 in South Washington County high schools. The calibrated model parameters were then adjusted to reflect the epidemiology of Omicron variants [24–28]. The model was used to simulate an Omicron wave during an 11 week fall trimester, simulating SARS-CoV-2 infections and learning loss on a daily basis under different isolation, quarantine and testing strategies and under best/worst case scenarios of mask usage and booster vaccine uptake.

### 2.1. Disease dynamics

**Figure 1.**
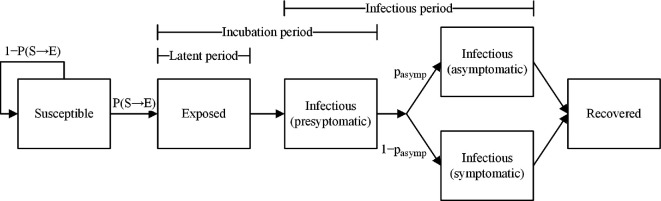
Health state transition diagram. In each daily time step, each agent may move between health states to reflect transmission (S→E), progression to infectiousness ($E\to I_{p}$), progression to symptomatic or asymptomatic infections ($I_{p}\to I_{a}$ or $I_{p}\to I_{s}$) and recovery ($I_{a}\to R$ and $I_{s}\to R$). An agent’s daily probability of infection, P(S→E), is calculated based on contact with infectious agents during the school day and applied as a fixed probability on weekends. Progression through the other health states is determined by state duration times, with some proportion of infections, $p_{asymp}$, being asymptomatically infectious. Model parameter values are listed in table 1.

The progression of COVID-19 was modelled in discrete time using the following health states: susceptible (S), exposed (E, infected but not infectious), infectious (I) and recovered (R, immune to infection). The infectious state was further subdivided into pre-symptomatic (I_p_), symptomatic (I_s_) and asymptomatic (I_a_) states. In the model, a susceptible individual enters the exposed state upon contracting COVID-19 from an infectious individual; after a fixed latent period, they transition to the pre-symptomatic infectious state, after which they then progress to either the symptomatic or asymptomatic infectious state. Once an individual is no longer infectious to others, they transition to the recovered state (figure 1). This COVID-19 model structure reflects the consensus that there is a delay from exposure to infectiousness and that infections are frequently asymptomatic. We also assume that infection confers immunity for the duration of the 11 week trimester time horizon. Transitions between health states were governed by the following disease progression parameters: latent period (time spent in E), incubation period (time spent in E and I_p_) and infectious period (time spent in I_p_ and I_s_ or I_a_). The latent and incubation periods were each fixed to an average value from the literature for all individuals [25,26], while the infectious period of each infection was randomly drawn from an empirical distribution derived from longitudinal sampling of a cohort of infected patients [27]. Disease progression parameter values and their corresponding source references are summarized in table 1. The population was also stratified by current quarantine/isolation status, vaccination status and mask usage in class, each of which modified an individual’s transmission and infection risks.

**Table 1.** Model parameters and data sources.

parameter	value	source
disease transmission		
hourly rate of transmission (delta)	0.0007 transmissions per contact per hour	calibration
increase in the rate of transmission of Omicron variant compared with delta variant	4.0	[24]
outside infection introduction rate (Fall 2022)	0.001 infections per weekend day per student	based on lowest rate in 2021
disease progression		
latent period (Omicron)	1 day	[25]
incubation period (Omicron)	3 days	[26]
infectious period (Omicron)	6.7 (1 to 15) days^a^	calculated from [27]
proportion of infections that are asymptomatic	0.405	[28]
rapid antigen test (RAT) characteristics		
sensitivity	0.63	[29]
surgical mask effectiveness		
inhale (infection rate reduction)	75%	[30,31]
exhale (transmission rate reduction)	50%	[30,32,33]
vaccine effectiveness (infection rate reduction)		
primary series, against Omicron	0	
booster dose, against Omicron	40%	[34,35]

^a^ Mean (minimum to maximum) values of an empirical distribution among Omicron-infected patients.

Transmission of SARS-CoV-2 was modelled through a network of contacts of 1954 students (size of a South Washington County high school) interacting in 6 one hour long class periods (averaging 25 students per class) and one 20 minute lunch period (each containing one-third of the student body). When susceptible and infectious students shared class or lunch periods, transmission was stochastically modelled as a Poisson process for each susceptible–infectious pair of students. The transmission rate of SARS-CoV-2 from an infectious student i to a susceptible student j present together in period, k, was calculated as follows:


$$r_{trans}\left(i,j,k\right)=\left(1-\delta_{\text{i}}^{m}\left(k\right)*RR_{mask\;exhale}\right)\left(1-\delta_{\text{j}}^{m}\left(k\right)*RR_{mask\;inhale}\right)\left(1-\delta_{\text{j}}^{V}*RR_{vaccine}\right)\lambda t_{k},$$

where *λ* is the hourly transmission rate and $t_{k}$ is the duration of the period k in hours (1 for class, one-third for lunch). The base transmission rate is reduced by preventative behaviours: ${RR}_{maskexhale}$ is the infection rate reduction achieved by filtering the exhaled aerosols of an infectious student, ${RR}_{maskinhale}$ is the infection rate reduction achieved by filtering the inhaled air by a susceptible student and ${RR}_{vaccine}$ is the infection rate reduction conferred to a susceptible student if they received that season’s COVID-19 vaccine. The indicator variables $\delta_{\text{i}}^{m}\left(k\right)$ and $\delta_{\text{i}}^{V}$ indicate whether a given student i masked during the period k and whether they received an updated COVID-19 vaccine dose, respectively. Note that during their lunch period, no student is masked, so $\delta_{\text{i}}^{m}\left(k\right)=0$ for all i when time period k reflects lunch. Rate reduction parameters were taken from the literature. Values and sources are summarized in table 1. The daily probability that a susceptible student j becomes infected (transitioning to the exposed state) is calculated as the sum of the transmission rates over the whole school day (six class periods plus one lunch period):


$$P_{j}\left(S\to E\right)=1-e^{-\sum_{k=1}^{7}\sum_{i\in I_{j}\left(k\right)}r_{trans}\left(i,j,k\right)}$$

where $I_{j}\left(k\right)$ is the set of infectious students present in student j’s kth period.

**Figure 2.**
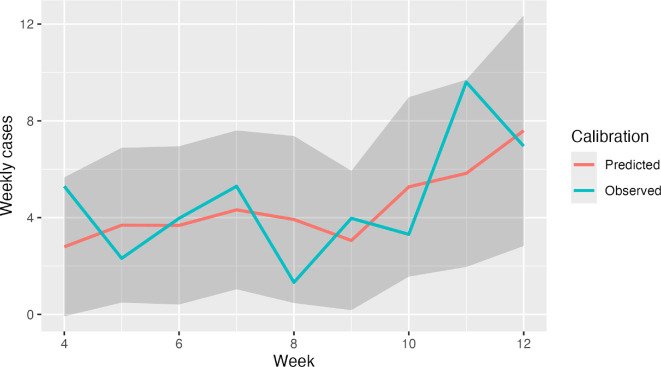
Plot of model behaviour when calibrated. Calibration target was weekly COVID-19 case counts for a South Washington County high school (blue), with 95% confidence band (grey shading) and mean model-predicted weekly cases (red), averaged over 500 simulations, using the best-fitting value of the hourly transmission rate parameter (0.0007). Other parameter values corresponded to the delta variant of COVID-19; see electronic supplementary material, table S1. Target data were based on aggregate COVID-19 case counts in three high schools reported by the South Washington County School District, rescaled to a single high school.

The hourly transmission rate, λ, was estimated for the delta SARS-CoV-2 variant through model calibration (see figure 2 and electronic supplementary material) and then scaled to reflect the relative increase in transmissibility of Omicron variants in our analysis [25]. In-school student contacts occurred only on weekdays (Monday–Friday), and students were excluded from the contact network if they were quarantining or isolating at home. Students could also be infected on the weekends (outside of school) with a risk proportional to community incidence. In base case simulations, outside infections were introduced on weekends at a constant rate of 100 infections per 1 00 000 per day, which was the lowest level of estimated community incidence during the fall 2021 calibration period (see electronic supplementary material). Minimal community incidence was chosen to focus the analysis on in-school infection dynamics and mitigation efforts; however, the community incidence rate was varied in sensitivity analysis.

### 2.2. Mitigation strategies

As a minimum baseline strategy, we assumed that any student with COVID-19 symptoms would conduct up to two rapid antigen tests (RATs), one at symptom onset and another 48 h later, in accordance with the latest United States Federal Drug Administration (FDA) guidance on the use of antigen tests [36]. Any student with a positive RAT would begin a 5 day isolation period at home. Two modifications to this strategy were considered: (i) issuing a 5 day quarantine for all classroom contacts of a confirmed case of COVID-19; and (ii) lengthening both the isolation and quarantine periods to 10 days. We also considered an intermediate isolation/quarantine policy, which involved 5 days of isolation/quarantine followed by 5 days of mask wearing at school (excluding lunch). These strategies reflect the range of CDC quarantine and isolation guidelines over the course of the pandemic [37].

**Figure 3.**
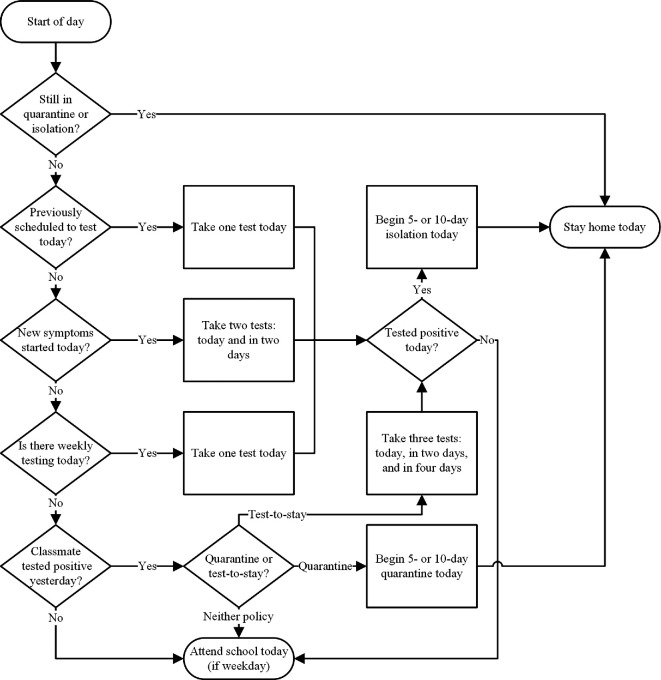
Flowchart for an individual agent’s behaviour on weekdays, showing whether they attend school (and are part of the contact network) or stay at home (removed from the network).

In combination with the different isolation and/or quarantine policies, we also considered two testing strategies: (i) proactive weekly testing of all students and (ii) reactive testing of classroom contacts of a confirmed case of COVID-19 used in lieu of quarantine (‘test-to-stay’) [38]. All testing was assumed to be done with RATs and included the possibility of false negatives based on RAT sensitivity estimates [19], which were varied in sensitivity analysis. Proactive testing consisted of a single weekly RAT of all students, while reactive testing consisted of a series of three RATs each 48 h apart for students with a known classroom exposure, consistent with FDA guidance for asymptomatic testing [36]. Figure 3 shows a simplified diagram of how these strategies modify agent behaviour.

### 2.3. Behavioural scenarios

Isolation, quarantine and testing strategies were evaluated in the context of different levels of two transmission-modifying behaviours: mask use and vaccine uptake. We considered scenarios of voluntary (10%) or mandatory (100%) mask use, assuming the inward and outward protective effectiveness of surgical masks [30–33] and varying mask effectiveness in sensitivity analysis. Masks only provided protection during class periods; students were assumed to be unmasked during lunch. In combination with masking, we also considered scenarios reflecting low (25%) or high (50%) uptake of COVID-19 booster vaccine doses, which were assumed to provide partial protection against Omicron infection [34,35].

### 2.4. Outcome measures

The model was used to simulate two primary outcome measures: the cumulative percentage of students who experienced a SARS-CoV-2 infection (a reflection of COVID-19 burden) and the average proportion of in-person learning days lost (a measure of learning loss burden) during an 11 week trimester. A day of lost in-person learning was incurred for each weekday a student spent at home due to COVID-19 isolation or quarantine.

## 3. Results

**Figure 4.**
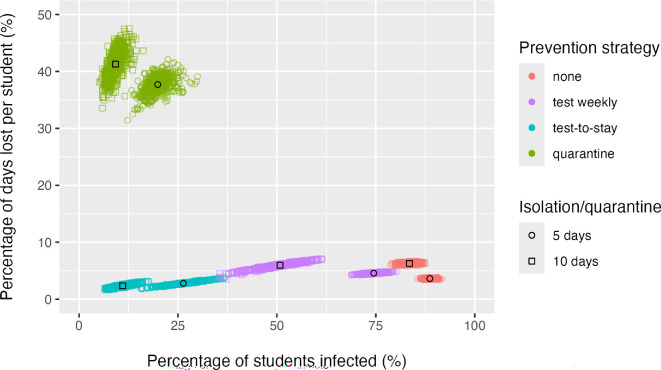
Learning loss versus infections for different mitigation strategies under a baseline scenario of voluntary mask use (10%) and booster uptake (25%). Each point reflects the simulated learning loss and infection outcome over an 11 week trimester for one of 500 stochastic simulations. Mean outcomes for each strategy are indicated by a black circle (for 5 day isolation/quarantine strategies) or black square (for 10 day isolation/quarantine strategies).

The calibrated model reproduced weekly case counts within 95% confidence intervals over the autumn 2021 trimester in South Washington County high schools (figure 2). Simulating outcomes for an Omicron-like variant over a subsequent autumn trimester with 10% of students voluntarily wearing masks and 25% having received a COVID-19 booster dose (baseline scenario), a policy of 5 or 10 day isolation alone resulted in the highest proportion of students infected (88.7 and 83.5%, on average, respectively; figure 4) with a moderate level of learning loss (an average of 3.6 and 6.3% days lost, respectively). The addition of quarantining close contacts for 10 days resulted in the fewest number of students infected (9.2% on average) but resulted in students losing a staggering 41.3% of in-person learning days. Reducing quarantine to 5 days increased infections but only achieved a small reduction in lost in-person learning days.

Using a test-to-stay strategy, reduced infections by almost as much as quarantine, but at far lower levels of learning loss. In fact, a test-to-stay strategy with a 10 day isolation period resulted in the fewest learning days lost (2.3% on average) and the lowest percentage of students infected (11% on average); a test-to-stay strategy with a 5 day isolation period had higher expected number of days of learning lost (2.8%) and students infected (26.4%) than with 10 day isolation, but still substantially lower than without testing or quarantine. Weekly testing of all students with a 10 day isolation period reduced both average learning loss and infections relative to isolation alone, though not to the same extent as test-to-stay. However, when combined with a 5 day isolation period, weekly testing resulted in fewer infections but higher learning loss than isolation alone.

**Figure 5.**
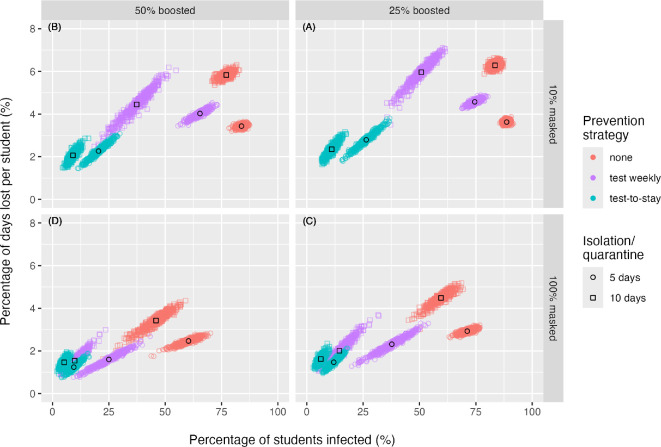
Learning loss versus infections of mitigation strategies across four scenarios of booster uptake and mask use. Panel (A): baseline scenario of 10% voluntary mask use and 25% booster dose uptake. Panel (B): scenario of higher booster uptake of 50% with 10% voluntary mask use. Panel (C): mask mandate scenario with 100% mask use and baseline 25% booster uptake. Panel (D): mask mandate scenario of 100% mask use combined with higher booster uptake of 50%. Each point reflects the simulated learning loss and infection outcome over a 11 week trimester for one of 500 stochastic simulations. Mean outcomes for each strategy are indicated by a black circle (for 5 day isolation/quarantine strategies) or black square (for 10 day isolation/quarantine strategies). The classroom quarantine strategies are omitted.

If uptake of booster vaccination was increased to 50%, infections and learning loss were slightly reduced for all strategies, but results remained qualitatively similar to the baseline scenario (figure 5B). Introducing a mask mandate (100% mask use) similarly reduced both infections and learning loss for all strategies, but with a stronger effect than increasing booster uptake (figure 5C). With high levels of masking, test-to-stay still resulted in the fewest infections and learning days lost. Weekly testing, while still not as effective as test-to-stay, reduced both infections and learning loss relative to isolation alone, regardless of the length of the isolation period. In a scenario with both the highest mask use and booster uptake levels, differences in learning loss and infections between strategies were markedly reduced (figure 5D). In this scenario, all strategies resulted in fewer than 5% of learning days lost; however, isolation-only strategies still resulted in 45.9–60.4% of students infected. For all testing strategies, lengthening the isolation period from 5 to 10 days reduced both infections and learning loss, and weekly testing with a 10 day isolation period achieved similar outcomes to test-to-stay strategies. Only under isolation alone did the longer 10 day isolation period increase learning days lost.

Compared with a 5 day isolation period, instituting a 5 day isolation period plus 5 days of required masking upon return to school resulted in a modest reduction in infections (electronic supplementary material, figure S1). This reduction was greater for the higher boost uptake scenario than the baseline scenario and also greater when isolation was combined with testing, indicating the synergistic effects of layered mitigation.

Sensitivity analyses on key model outcomes (transmission rate, community incidence, test sensitivity, mask effectiveness and isolation/quarantine adherence) are presented in electronic supplementary material, table S3 and figures S2–S6. In general, trade-offs between learning loss and infection prevention under mitigation strategies arose more often when conditions were unfavourable, such as greater community incidence, lower test sensitivity, lower mask effectiveness and lower adherence to isolation and testing requirements. Mask mandate scenarios were more resilient to unfavourable conditions in preserving testing strategies’ infection and learning loss benefits relative to isolation alone. Strategy outcomes under the mask mandate scenario were sensitive to mask type. A mask mandate always reduced both infections and learning loss compared with voluntary mask use for any strategy, but reductions were most pronounced when assuming the use of N95 respirators. However, even universal use of cloth masks resulted in modest benefits, indicating that school mask mandates may be beneficial even if N95 respirators are not universally used.

## 4. Discussion

Like other modelling studies of COVID-19 mitigation in schools, we find that quarantining close contacts reduces infections but is highly disruptive to in-person learning, while testing can achieve similar reductions in infections with at most a small cost to learning. We also find that lengthening the isolation period further mitigates infections without increasing in-person learning loss as long as there are sufficient complementary mitigation measures in place, such as post-exposure testing or weekly testing with high levels of mask use.

While much of the debate on COVID-19 policies in schools has framed the cost of more stringent COVID-19 mitigations as an increase in learning loss, COVID-19 infections themselves lead to learning loss while a student is out sick. Our study highlights that trade-offs exist when a mitigation measure increases exclusion from in-person learning without achieving a sufficient reduction in infections and associated infection-related learning loss (see electronic supplementary material for a mathematical derivation). For example, quarantining close contacts achieves a large reduction in infections, but at a loss of over 40% of in-person learning days because it relies on keeping all exposed students home, many of whom are not infected. In contrast, test-to-stay involves far less learning loss because exposed students remain in class and repeat testing identifies enough infections early enough to avert a similar number of infections as a full quarantine. Similarly, increasing the isolation period from 5 to 10 days doubles the learning loss associated with each infection. While a longer isolation period does avert more infections because many students are still infectious past 5 days, without any additional prevention measures, the net result is an increase in learning loss, largely from failing to mitigate pre-symptomatic and asymptomatic transmission.

In our study, we highlight conditions under which averting COVID-19 infections comes at a cost of increased learning loss; however, we are not evaluating the optimal balance between infection risk and learning loss. The near-term learning loss of any school-based COVID-19 mitigation policy must be weighed against the growing literature on the medium- and long-term impacts of COVID-19 infection in children and adolescents, including non-negligible risks of long COVID [39]. Importantly, in this analysis, we only capture the in-person learning loss due to isolation or quarantine periods; we do not consider the potential for protracted illness or symptoms, including cognitive impairments, lingering fatigue or increased susceptibility to other illnesses, that may also interfere with effective learning.

Like other studies, we find that a layered mitigation approach involving isolation, testing, masks and vaccination is most effective at reducing infections, as well as learning loss. When other measures are in place to reduce the risk of infection, such as a mask mandate or high levels of booster dose uptake, trade-offs between learning loss and preventing infections are reduced or eliminated. This was particularly true for weekly surveillance testing strategies, the effectiveness of which is heavily dependent on the risk of transmission in the inter-test interval before newly infected individuals can be identified and isolated. When the transmission is lowered, such as through increases in mask use and/or booster uptake, surveillance testing becomes both more effective and less costly in terms of learning loss, making it a viable alternative to the (logistically more complex) test-to-stay strategies.

The simulation model used in this study is necessarily a simplification of complex dynamic processes and has several limitations. First, disease dynamics were largely homogenous, with all students having the same incubation and pre-symptomatic periods and being equally infectious over the course of their illness, though duration of infectiousness was stochastically sampled from a distribution. Tests were assumed to have a constant sensitivity over the entire infectious period and across symptomatic and asymptomatic students. The contact network was also simplified, with an assumption of a fully randomized class schedule, ignoring clustering by student characteristics like grade level and subject interests. The number of lost in-person days is also a simplified metric of learning loss. Missed school days have different effects for each student. It also does not reflect how learning loss is distributed among students or whether some students bear a disproportionate burden. A final caveat is that some simulated realizations may have reached unrealistically high levels of illness and absenteeism that would have interfered with normal school functioning and likely necessitated a transition to virtual learning, as ocurred in many schools during the first Omicron wave in early 2022.

Despite these limitations, our modelling study provides insight into when and how trade-offs exist between COVID-19 mitigation and learning loss. Our findings demonstrate that an effective, layered mitigation approach can avoid exacerbating learning loss and lower the risk of infection in educational settings. As schools continue to be challenged by COVID-19, this model generates evidence that can aid decision-makers and inform the ongoing discussion around COVID-19 mitigation in schools.

## Data Availability

Model simulation results and analysis code for this research work are stored in [40] and have been archived within the Zenodo repository: [41]. Supplementary material is available online [42].
